# *In vitro* exposure to *Hymenoptera* venom and constituents activates discrete ionotropic pathways in mast cells

**DOI:** 10.1080/19336950.2019.1629225

**Published:** 2019-06-25

**Authors:** C. Jansen, L. M.N. Shimoda, J. Starkus, I. Lange, N. Rysavy, K. Maaetoft-Udsen, C. Tobita, A.J. Stokes, H Turner

**Affiliations:** a Laboratory of Immunology and Signal Transduction, Division of Natural Sciences and Mathematics, Chaminade University, Honolulu, Hawai‘I, USA; b Department of Pharmaceutical Sciences, Daniel K. Inouye College of Pharmacy, University of Hawai‘i at Hilo, Hilo, Hawai‘i, USA; c Department of Cell and Molecular Biology, Laboratory of Experimental Medicine, John A. Burns School of Medicine, University of Hawai‘i, Honolulu, Hawai‘i, USA

**Keywords:** Mast cells, calcium, bee venom, allergy

## Abstract

Calcium entry is central to the functional processes in mast cells and basophils that contribute to the induction and maintenance of inflammatory responses. Mast cells and basophils express an array of calcium channels, which mediate responses to diverse stimuli triggered by small bioactive molecules, physicochemical stimuli and immunological inputs including antigens and direct immune cell interactions. These cells are also highly responsive to certain venoms (such as *Hymenoptera* envenomations), which cause histamine secretion, cytokine release and an array of pro-inflammatory functional responses. There are gaps in our understanding of the coupling of venom exposure to specific signaling pathways such as activation of calcium channels. In the present study, we performed a current survey of a model mast cell line selected for its pleiotropic responsiveness to multiple pro-inflammatory inputs. As a heterogenous stimulus, *Hymenoptera* venom activates multiple classes of conductance at the population level but tend to lead to the measurement of only one type of conductance per cell, despite the cell co-expressing multiple channel types. The data show that I_CRAC_, I_ARC,_ and TRPV-like currents are present in the model mast cell populations and respond to venom exposure. We further assessed individual venom components, specifically secretagogues and arachidonic acid, and identified the conductances associated with these stimuli in mast cells. Single-cell calcium assays and immunofluorescence analysis show that there is heterogeneity of channel expression across the cell population, but this heterogeneity does not explain the apparent selectivity for specific channels in response to exposure to venom as a composite stimulus.

## Introduction

Calcium is a central second messenger to a plethora of biological processes [1]. Calcium entry in mast cells and basophils regulates cytokine gene transcription, secretory granule exocytosis, and other functional responses [2]. Calcium fluxes across the plasma membrane in mast cells and basophils are conveyed by a markedly diverse set of calcium channels, including calcium release activated calcium (CRAC) channels, arachidonic acid-activated channels (ARC), various transient receptor potential (TRP) channels of the C (canonical), M (Melastatin-like), and V (Vanilloid) sub-families. KIR, KCa as well all of the different Kv potassium channels have also been reported in mast cells and basophils [3]. These support calcium entry through modulating driving force (membrane potential) in a receptor-driven and immunologically responsive manner [4]. With so many parallelized calcium entry pathways across the PM, cells clearly have the potential to respond to diverse single inputs.

Stimuli that induce calcium mobilization in mast cells and basophils include immunological stimuli (innate and adaptive immune pathways including Toll-like Receptors (TLR) and high-affinity receptor for IgE (FcεRI) and Fcγ receptor signaling). Physical and mechanical stimuli, and potent small molecules from diverse chemical families (including arachidonic acid, various plant-derived and other environmental stimuli, etc.) also activate mast cells and basophils. *Hymenoptera* venoms are potent activators of mast cells and basophils, inducing secretory granule exocytosis and histamine release. Envenomation sites display *rubor, calor, dolor*, and *tumor* [5], primarily orchestrated by the release of factors that induce inflammation, activate sensory neurons, increase vascular permeability and recruit other leukocytes using chemokine gradients. The components of venoms are incompletely understood (no complete proteome and metabolome have yet been published), but the classes of mediator that they contain include antigens, peptide and small-molecule secretagogues, and soluble phospholipase A2 (which generates arachidonic acid at the plasma membrane). Example published compositions of venoms include a cocktail of many proteins and enzymes, which include hyaluronidase, phospholipases (PLA) A1 and A2, mellitin, mastoparans, apamine, mast cell degranulating peptide, histamine, adolapin, oligopeptides, phospholipids, saccharides, acid phosphatase, and histamine [6]. Clearly, venoms are a complex, multi-component, stimulus that, when impacting the mast cell or basophil membrane, would have several co-incident mechanisms to activate multiple calcium entry pathways in parallel. The current study has two motivations. First, bee venoms are associated with human pathology ranging from irritation to anaphylaxis, and ion channels activated by the venoms are intrinsically targets for possible intervention. However, in order to intervene, it is necessary to know which ion channels should be targeted and are relevant to envenomation. Conversely, bee venoms and their components are being proposed as therapies in diverse disorders [7]. Again, an understanding of their mechanisms of action is critical to predicting therapeutic modality and potential side effect profiles. One of the motivations for the current study is, therefore, the identification of the specific ionotropic responses initiated by bee venom in a model mast cells. Second, in the Ca^2+^ signaling field, stimuli that couple to ion channels tend to be studied unilaterally and in isolation. Perhaps less well-understood are the pathways and impact of co-incident stimuli that could potentially activate multiple calcium entry routes simultaneously, an example of which would be *Hymenoptera* envenomation.

In the current study, we dissected the calcium responses to venom and examples of its components, with a particular interest in assessing whether disparate calcium entry pathways were simultaneously activated or whether individual pathways took precedence. Three entry pathways were the focus of this study, although there is undoubtedly more diversity of channel expression in these cells [8–12].

First, calcium release activated calcium (CRAC) currents were first described in the early 1990s in mast cells and chromaffin cells [13]. The molecular identity for I_CRAC_ is a hexamer of ORAI1 subunits [14,15]. CRAC channels are highly calcium selective and are inhibited by Lanthanum and 2-Aminoethoxydiphenyl borate (2-APB) [16]. CRAC channels are activated by endoplasmic reticulum (ER) calcium store depletion, which leads to store-operated calcium entry (SOCE). The activation of CRAC channels occurs when stromal interaction molecule 1 (STIM1), a calcium sensor in the ER, moves to the membrane and directly activates ORAI1 channels [17,18]. CRAC channel current-voltage relationships show a high level of inward rectification and reverse near +60 mV.

Second, Transient Receptor Potential Vanilloid channels (TRPV family) are nonselective cation channels that are largely associated with pain sensation [19], and a number of the family members are expressed in mast cells. TRPVs in mast cells respond to further physicochemical stimuli and a range of small molecules. For example, TRPV1 is activated by capsaicin, heat, anandamide, and exogenous cannabinoids [20]. Activation of TRPV1 by the venom peptide mellitin [21,22] has been described for TRPV1 in sensory neurons but not in mast cells [23,24]. TRPV1 is permeable to the cations sodium, lithium, potassium, cesium, calcium, and magnesium [25], and is not inhibited by Lanthanum or 2-APB. The current-voltage relationship for TRPV1 shows an outwardly rectifying current that reverses near 0 mV.

Third, arachidonic acid-regulated channels (ARC) are also a source of calcium entry into the mast cells [26,27]. ARC channels share similar biophysical characteristics with CRAC channels in that ARC channels are highly selective for calcium and have small conductances similar to CRAC channels. ARC is composed of ORAI1 and ORAI3 subunits [28]. The ARC current-voltage relationship is very similar to CRAC channels, showing positive reverse potential near +60 mV and a high inward rectification [29]. Differentiation between ARC and CRAC requires analysis of sensitivity to the CRAC (but not ARC) inhibitor 2-APB.

In the current study, we exposed a model mast cell line to venoms, and individual venom components or products. We sought to assess the impact of complex venoms on activation of calcium entry pathways to understand further the decoding of these multi-component stimuli and to identify the calcium entry mechanisms that would hold subsequent importance for understanding venom pathology and outcomes/mechanism of therapeutic venom exposure [30]. We performed a current survey focusing on these three calcium entry mechanisms of a model mast cell line selected for its pleiotropic responsiveness to multiple pro-inflammatory inputs. The data show that I_CRAC_, I_ARC_, and TRPV conductances are present in model mast cell populations, but at varying frequencies and abundancies, and responding to diverse single stimuli. *Hymenoptera* venom acts as a composite stimulus and activates multiple classes of conductance at the population level but tend to lead to the induction of only one type of conductance per cell.

## Methods

### Cell culture

RBL2H3 [31] were grown at 37°C, 5% CO_2_, in 95% humidity in Dulbecco’s Modification of Eagle Medium (Mediatech Inc., Herndon, VA) with 10% heat-inactivated Fetal Bovine Serum (Mediatech) and 2 mM L-glutamine. HEK TRexTRPV1 were cultured in DMEM, 10% Fetal Bovine Serum, 2 mM L-glutamine, 10 μg/ml Blasticidin (Calbiochem, San Diego CA), 400 μg/ml Zeocin (InvivoGen, San Diego CA), transgene expression was induced using 1 μg/ml Tetracycline for 16–24 h.

### Chemicals, reagents, and stimulations

General chemicals were from VWR (West Chester, PA) and Sigma Aldrich (St. Louis, MO). Phorbol myristate acetate (PMA) and Ionomycin were from Calbiochem (Gibbstown, NJ). IgE anti-DNP is from Sigma and KLH-DNP was from Calbiochem. Bee venom was from HollisterStier (Spokane, WA). To mitigate batch-to-batch variation in venom, three independent batches were selected on the basis of similar potency for induction of histamine release in control experiments, mixed, aliquoted and used for the duration of the studies presented here. Mastoparan and Mellitin were from Sigma Aldrich. Arachidonic acid was from Enzo (Farmingdale, NY). 2-Aminoethoxydiphenyl borate (2-APB) was from Calbiochem (La Jolla, CA). Capsaicin and Capsazepine were from Sigma Aldrich. FcεRI stimulation used 0.1 μg/ml IgE anti-DNP for 16 h at 37°C, followed by three washes and the addition of 250 ng/ml KLH-DNP for the indicated times. PMA and ionomycin were both used at 500 nM. Arachidonic acid and other stimuli were used at concentrations specified in the Figure legends. Antibodies were obtained as follows: ORAI1: rabbit polyclonal (Abcam, Cambridge, UK), mouse monoclonal (ThermoFisher, Waltham, MA); ORAI3, rabbit polyclonal (Abcam), mouse monoclonal (ThermoFisher); TRPV1, rabbit polyclonal AJS/HT, mouse monoclonal BS397 (Abcam); TRPV2 rabbit polyclonal AJS/HT, anti-TRPV2-FITC rabbit polyclonal directly conjugated (Alomone); TRPA1 rabbit polyclonal (AJS, Bethyl; Laboratories), mouse monoclonal (Abnova). Secondary antibodies were Alexa-488 or 568 goat anti-rabbit or rabbit anti-mouse (Molecular Probes, Eugene, OR).

### Mast cell degranulation assay

RBL2H3 were plated in 48 well cluster plates at 5 × 10^4^ cells/well. Cells were primed for 16 h at 37°C with 1 µg/ml IgE anti-dinitrophenol (DNP, Sigma, St Louis, MI). Monolayers were washed and incubated in 200 µl Tyrode’s buffer before stimulating as described in the Figure legend. After 45 min at 37°C, 25 µl supernatant was removed, clarified by microcentrifugation, and transferred to a 96-well plate containing 100 µl per well PNAG substrate solution (1 mM p-N-acetyl glucosamine (Sigma) in 0.05 M citrate buffer pH 4.5). After 1 h at 37°C, reactions were quenched by the addition of 100 µl per well 0.2 M glycine, pH 9.0. Beta-hexosaminidase levels were read as OD at 405 nm. Results are shown as the mean ± standard deviation.

### Calcium assay (bulk method)

RBL2H3 were washed and incubated with 0.2 μM Fluo-4 [32] for 30 min at 37°C in a modified Ringer’s solution of the following composition (in mM): NaCl 145, KCl 2.8, CsCl 10, CaCl_2_ 10, MgCl_2_ 2, glucose 10, Hepes·NaOH 10, pH 7.4, 330 mOsm. Nominally calcium-free conditions were generated by omitting CaCl_2_ and adding 2mM EGTA. Cells were transferred to 96-well plates at 100,000 cells/well and stimulated as indicated. Calcium signals were acquired using a Flexstation 3 (Molecular Devices, Sunnydale, USA). Data were analyzed using SoftMax® Pro 5 (Molecular Devices). Where indicated, nominally calcium-free external conditions were achieved by the preparation of 0 mM CaCl_2_ Ringer solution containing 1 mM EGTA.

### Calcium assay (single cell method)

RBL2H3 were plated on glass coverslip dishes (MatTek, Ashland, MA) and incubated with 1 µM Fluo-4 for 30 min at 37°C in modified Ringer’s solutions as described above. After washing, cells were stimulated as indicated on a 37°C heated stage. Calcium signals were acquired using a Nikon Ti Eclipse confocal microscopy system, using EZ C1 software for acquisition and NIS Elements software (Nikon) for analysis. Where indicated, nominally calcium-free external conditions were achieved by the preparation of 0 mM added CaCl_2_ Ringer solution containing 1 mM EGTA.

### Electrophysiology

Cells grown on glass coverslips were transferred to the recording chamber and kept in a standard-modified Ringer’s solution. For the experiments measuring *I*_CRAC_ and *I*_ARC_ like currents a standard-modified Ringer’s external solution of the following composition was selected (in mM): NaCl 120, CaCl_2_ 20, MgCl_2_ 2, CsCl 10, glucose 10, Hepes·NaOH 10, pH 7.2, with osmolarity typically ranging from 295 to 325 mOsm. Intracellular pipette-filling solutions contained (in mM): Cs-glutamate 120, MgCl_2_ 1, CaCl 4, Cs-BAPTA 10, HEPES·CsOH 10, pH 7.2 adjusted with CsOH with a free calcium concentration of 150 nM. The external solutions used with the TRPV1 overexpressing HEK cells contained: NaCl 140, KCl 2.8, CaCl_2_ 1, MgCl_2_ 2, Hepes·NaOH 10. The internal solution used with TRPV1 overexpressing HEK cells contained: Cs-glutamate 120, Cs Bapta 10, CaCl_2_ 4.1, MgCl_2_ 1, NaCl 8, HEPES·CsOH 10, pH 7.3 and osmolarity around 300 mOsm. The internal solution contained 150 nM free calcium. Patch-clamp experiments were performed in the tight-seal whole-cell configuration at 21–25°C. High-resolution current recordings were acquired by a computer-based patch-clamp amplifier system (EPC-10-USB, HEKA, Lambrecht, Germany). Patch pipettes had resistances between 2 and 4 MΩ after filling with the standard intracellular solution. Immediately following the establishment of the whole-cell configuration, voltage ramps of 50 ms duration spanning the voltage range of – 100 to +100 mV were delivered from a holding potential of 0 mV at a rate of 0.5 Hz over a period of 500 s. All voltages were corrected for a liquid junction potential of 10 mV between external and internal solutions because of glutamate use as an intracellular anion. Currents were filtered at 2.9 kHz and digitized at 100 µs intervals. Capacitive currents and series resistance were determined and corrected before each voltage ramp using the automatic capacitance compensation of the EPC-10-USB. The current development graphs were generated by extracting currents at −80 mV and +80 mV. Where applicable, statistical errors of averaged data are given as means ± SEM with n determinations. Trace subtraction was performed on small calcium currents to measure *I*_CRAC_ and *I*_ARC_ like currents. A stable trace was selected and subtracted from all of the traces, which removes the current recorded prior to application leaving only current that develops after application of the compounds. If internal solution application was used, the earliest stable trace was selected for subtraction.

### Imaging

Immunofluorescence staining was carried out as follows: Cells were seeded on glass coverslips and transferred to 24-well cluster plates for staining. Fixation was with methanol (1 min). Blocking was with 5% (v/v) fish skin gelatin (FSG) for 15 min at RT. Primary antibodies were applied singly or in combination in PBS 0.1% (v/v) FSG for 1 h. After three washes in PBS, secondary antibodies were applied at 0.1 μg/ml for 30 min in PBS 0.1% (v/v) FSG. After three washes in PBS, coverslips were dipped in dH_2_O and mounted in Crystal Mount (Electron Microscopy Services). Secondary-alone coverslips were prepared with a sham exposure (no primary antibody exposure), but all other steps in the protocol unchanged. Bright field and fluorescence imaging of cells were performed on a Nikon Ti Eclipse C1 epi-fluorescence and confocal microscopy system. Images were analyzed in NIS Elements (Nikon, Melville, NY). Unless otherwise stated, images were acquired through a Plan Apo VC 100 × 1.40 oil objective (Nikon).

### Analysis

Results are shown as the mean ± standard deviation. Statistical significance was determined based on Student’s t-test or ANOVA. Adjacent to data points in the respective graphs, significant differences were recorded as follows: single asterisk, p < 0.05; double asterisk, p < 0.01; triple asterisk, p < 0.001; no symbol, p > 0.05. Experiments are all *n* of at least 3.

## Results

### Bee and wasp venoms differentially induce functional responses in model mast cells

**Figure 1.**
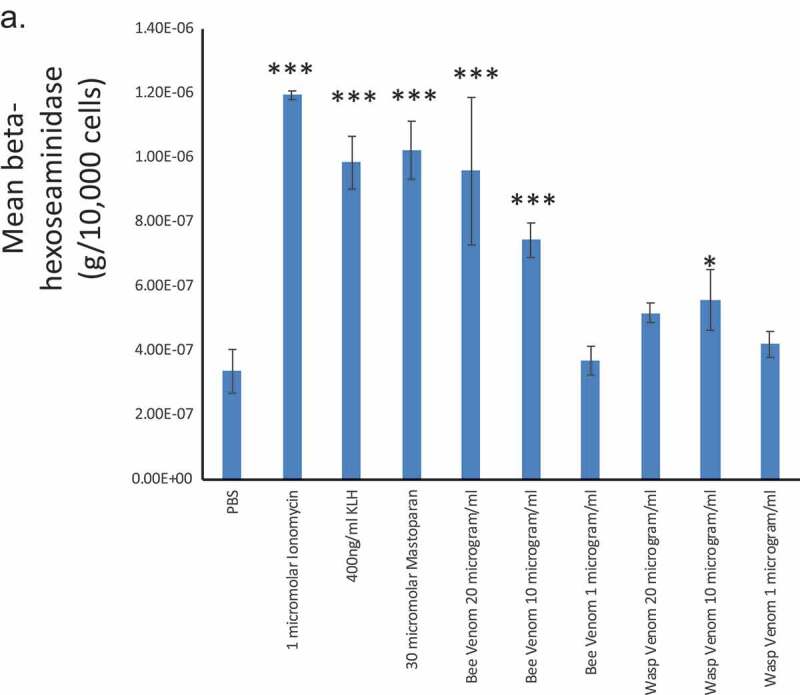
(a) Beta-hexosaminidase release from RBL2H3. RBL2H3 were stimulated for 60 min with the indicated agents and levels of beta-hexosaminidase secreted into the extracellular milieu were assayed using a colorimetric protocol. Data are presented as standard errors around a mean of triplicate samples, and significant (Student’s t-test) differences from non-stimulated (NS) controls are denoted by asterisks.

Bee and wasp venoms both activate mast cell-driven inflammatory responses. Both contain potentially ionotropic and calcium-mobilizing stimuli which contain numerous components including peptide antigens, secretagogues, arachidonate-generating enzymes, and small molecules. We identified sources of both venoms and obtained multiple batches that were then mixed and aliquotted to provide long-term consistency across the experiments presented in this study. We initially screened doses of bee and wasp venoms for the ability to induce degranulation in the model mast cell line selected for this study (Figure 1). Our dose-response analysis showed that lytic events are uncommon (trypan blue positivity after 30 μg/ml venom exposure for 25 min <15% in both venoms), and that secretory events are observed in response to both venoms. Figure 1 shows a beta-hexosaminidase assay (a proxy for histamine release), which was performed on the RBL2H3 cells after 30 min of stimulation by both wasp venom and bee venom. Both venoms showed the release of beta-hexosaminidase in a dose-dependent manner. The lower potency of wasp venom was noted and in subsequent experiments, we focused on bee venom. The wasp venom showed a level of histamine release that was lower than the mastoparan, a secretagogue peptide in wasp venom, as well as ionomycin. The bee venom showed a dose-dependent increase in histamine release that approached that achieved through ionomycin.

### Bee venom initiates Ca^2+^ entry and diverse Ca^2+^-permeant conductances in mast cells

**Figure 2.**
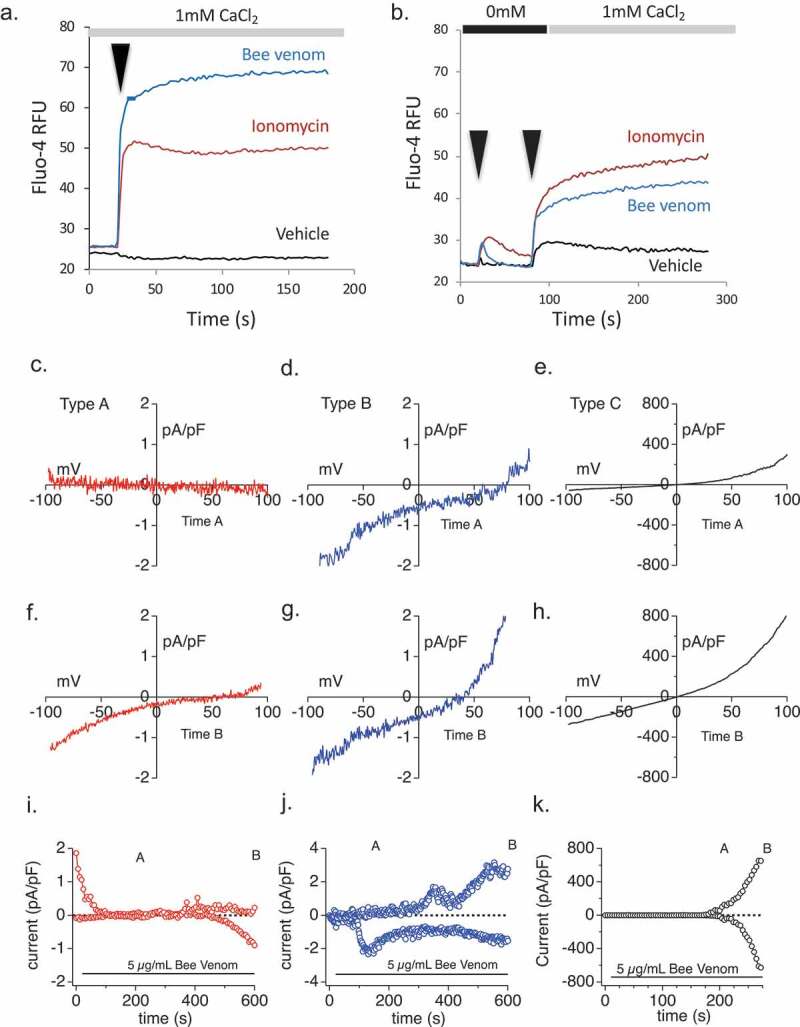
Venom-induced Ca^2+^ responses and conductances in RBL2H3. (a,b) Population-based calcium assays of RBL2H3 responses to venom and venom-associated stimuli in the presence and absence of external calcium. Fluo-4-AM was used to load RBL2H3 in a modified Ringer’s solution containing 1 mM external CaCl_2_. Experimental readings were carried out either in 1mM external CaCl_2_ (a) or in 0mM CaCl_2_ with 1mM EGTA followed by re-addition of CaCl_2_ to a concentration of 1mM final (b). Cells were stimulated after a period of establishing baseline (usually 20 s) by addition of vehicle (matched diluent to each stimulus), Ionomycin (500 nM), or Bee Venom (5 µg/ml). (c–k) Bee venom current survey. (c,f,i) Type A representative current-voltage relationships taken after background subtraction at indicated time points (see current development graph in 2 I) of 5 µg/ml bee venom application. The data in Figures (c,f,i) were all recorded from the same RBL2H3 cell (red). (d,g,j) Type B current-voltage relationship taken after background subtraction at indicated time points (see current development graph in 2 J) of 5 µg/ml bee venom application. The data in Figure 2(d,g,j) were all recorded from the same RBL2H3 cell (blue). (e,h,k) Type C current-voltage relationships taken at indicated time points (see current development graph in 2 K) of 5 µg/ml bee venom application. The data in Figure 2(e,h,i,k) were all recorded from the same RBL2H3 cell (black). Note that application times were the same for each figure, but the extraction point for I/V curves may vary. The current development graphs were generated by extracting currents at −80 mV and +80 mV.

We applied bee venom (BV) to the RBL2H3 mast cell and measured both Ca^2+^ influx and the resulting conductances. Figure 2(a) shows that BV induces a large mobilization of intracellular Ca^2+^. Figure 2(b) dissects this response into the release from intracellular stores and influx. Cells were stimulated in a nominally calcium-free Ringer solution (0 mM additional CaCl_2_ plus 2 mM EGTA). Under these conditions, any calcium fluxes observed come from intracellular store depletion. At 100 s, extracellular calcium levels are restored to 1 mM, and any channels that have been opened either in response to store depletion or due to direct channel activation of non-store-operated channels, manifest as a large influx signal. We note that bee venom induces some store release and activates a large influx response.

The whole-cell patch-clamp technique was used to further investigate the effects of bee venom at the single-cell level in RBL2H3 cells. We performed a current survey where RBL2H3 cells were assayed in the whole-cell patch-clamp configuration and once the current-stabilized venom was applied. Across the cell population, we noted that different cells exhibited distinct conductances in response to this treatment. We preliminarily categorized these currents into three types – types A, B, and C. Type A, B, and C currents occurred in 50%, 33%, and 17% of the cells examined, respectively. The leak subtracted type A current (Figure (2c,f,i), red traces) stabilized after a few seconds and took about 500 s of bee venom application for current to activate. The type A current resulting from bee venom application is inward rectifying with a positive reverse potential. The Type A inward current at −80 mV showed on average 1 pA/pF current density. The leak subtracted type B current (Figure 2(d,g,j) blue traces) also stabilized after a few seconds, but the inward current appeared to activate faster at about 300 s as well as a contained a small outward component that inactivates over the 600 s. The current-voltage relationship for Type B shows a similar inward rectification as Type A but also contains a further positively shifted reverse potential. The type B current density measured at −80 mV showed on average around 1.2 pA/pF. The type C (Figure 2(e,h,k) black traces) current developed after 200 s of bee venom application and was much larger than the type A and type B currents. The type C current-voltage relationship shows an outwardly rectifying current that ends as a linear current with ~650 pA/pF of inward and outward current. The outward current developed more slowly than the inward current.

### The diverse venom-induced conductances can be attributed to I_ARC_, I_CRAC_ and TRPV1

**Figure 3.**
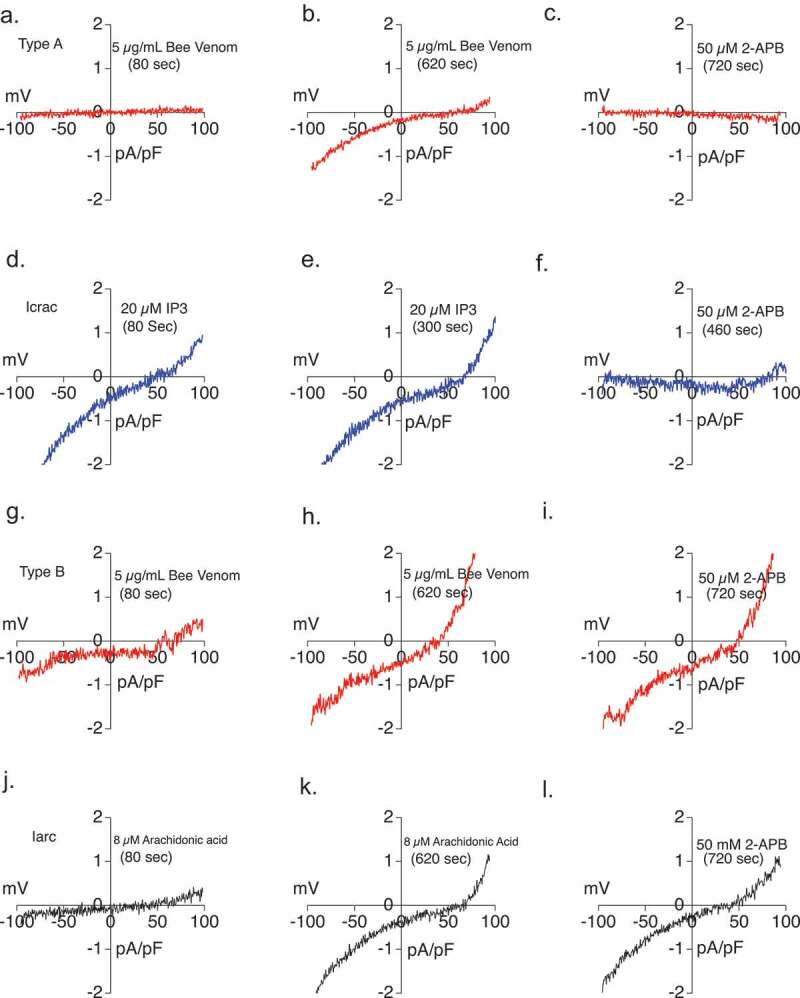
Attribution of CRAC- and Arc-like bee venom-induced currents using responsiveness to 2-APB. (a) Type A current-voltage relationship taken after background subtraction at 80 s of 5 µg/ml bee venom application. The data in Figure 3(a–c) were all recorded from the same RBL2H3 cell over time. (b) Type A current-voltage relationship taken after background subtraction at 620 s of recording and 600 s of 5 µg/ml bee venom application. (c) Current-voltage relationship recorded after background subtraction at 720 s of recording and 100 s of 50 µM 2-APB application to fully developed type A bee venom current. (d) *I*_crac_ current-voltage relationship taken after background subtraction at 80 s of 20 µM IP_3_ internal perfusion. The data in Figure 3(d,e,f) were all recorded from the same RBL2H3 cell (N = 5). (e) *I*_crac_ current-voltage relationship taken after background subtraction at 300 s of 20 µM IP_3_ internal perfusion. (f) Current-voltage relationship recorded after background subtraction at 460 s of recording with 100 s of 50 µM 2-APB application to fully developed *I*_crac_ current. (g) Type B current-voltage relationship taken after background subtraction at 80 s of 5 µg/ml bee venom application. The data in Figure 3 G, H and I were all recorded from the same RBL2H3 cell. (h) Type B current-voltage relationship taken after background subtraction at 620 s of recording and 600 s of 5 µg/ml bee venom application. (i) Current-voltage relationship recorded after background subtraction at 720 s of recording and 100 s of 50 µM 2-APB application to fully developed type B bee venom current. (j) *I*_arc_ current-voltage relationship taken after background subtraction at 80 s of 8 µM Arachidonic acid external application. The data in Figure 3(j,k,l) were all recorded from the same RBL2H3 cell (N = 4). (k) *I*_ARC_ current-voltage relationship taken after background subtraction at 620 s of recording and 600 s of 8 µM Arachidonic acid external application. (l) Current-voltage relationship recorded after background subtraction at 720 s of recording and 100 s of 50 µM 2-APB application to fully developed *I*_ARC_ current.

We sought to identify the ion channel supporting each of the Type A, B, and C conductances. Initially, we focused on Type A and B currents, which we hypothesized to be CRAC or ARC, which require some pharmacological approaches to differentiate them. Both have positive reversal potentials but CRAC is sensitive to 2-APB inhibition while ARC is not, a property that we used to differentiate them. The type A current which shows a ~+70 mV reverse potential and inward rectification was allowed to develop and then 50 µM 2-APB was applied to the Type A current. The 50 µM 2-APB application inhibited the type A current (Figure 3(a–c)). The Type B current did not show 2-APB inhibition (Figure 3(g,h,i)). Intracellular inositol (1,4,5) triphosphate (IP3) and extracellular arachidonic acid were applied to provide positive control recordings for CRAC and ARC, respectively (Figure 3(d,e,f,j,k,l)). The IP3 induced CRAC currents showed an inward rectifying current with ~ +70 mV reverse potential and sensitivity to 2-APB (Figure 3(d,e,f)). The arachidonic acid-induced ARC current also showed ~ +70 mV reverse potential and inward rectification. ARC was resistant to 2-APB inhibition (Figure 3(j,k,l)) but could be inhibited by the trivalent ion Lanthanum (La^3+^) (data not shown)

The type C current resembles a non-selective cation current of the TRP family. In Figure 4 we tested the hypothesis that the Type C current could be TRPV1. This hypothesis was based on the fact that one prior study in sensory neurons suggested that TRPV1 could be activated by the BV component Mellitin [23]. We first compared this current to TRPV1 and then applied a known TRPV1 inhibitor to the Bee venom-induced Type C current. HEK293 cells overexpressing TRPV1 (HEK V1) were used to investigate if bee venom can activate TRPV1. Figure 4 shows a validation of this experimental system. First, 10 µM Capsaicin, a TRPV1 agonist, was applied to wild-type HEK293 and HEKV1 cells as a control. Figure 4(a) shows that capsaicin-induced calcium responses are present in HEK expressing TRPV1 but not wild-type cells, validating the system. Similarly, Figure 4(b,c) show that capsaicin-induced conductances are present in HEK expressing TRPV1 but not wild-type cells. Figure 4(d,e) show that the BV-induced conductance develops in HEK-TRPV1 but not WT HEK. At the 5 μg/ml dose of BV, there is a long lag phase in the current development (Figure 4(e)), bit at higher doses the current develops along a similar time course to that with Capsaicin (Figure 4(f,g)).

**Figure 4.**
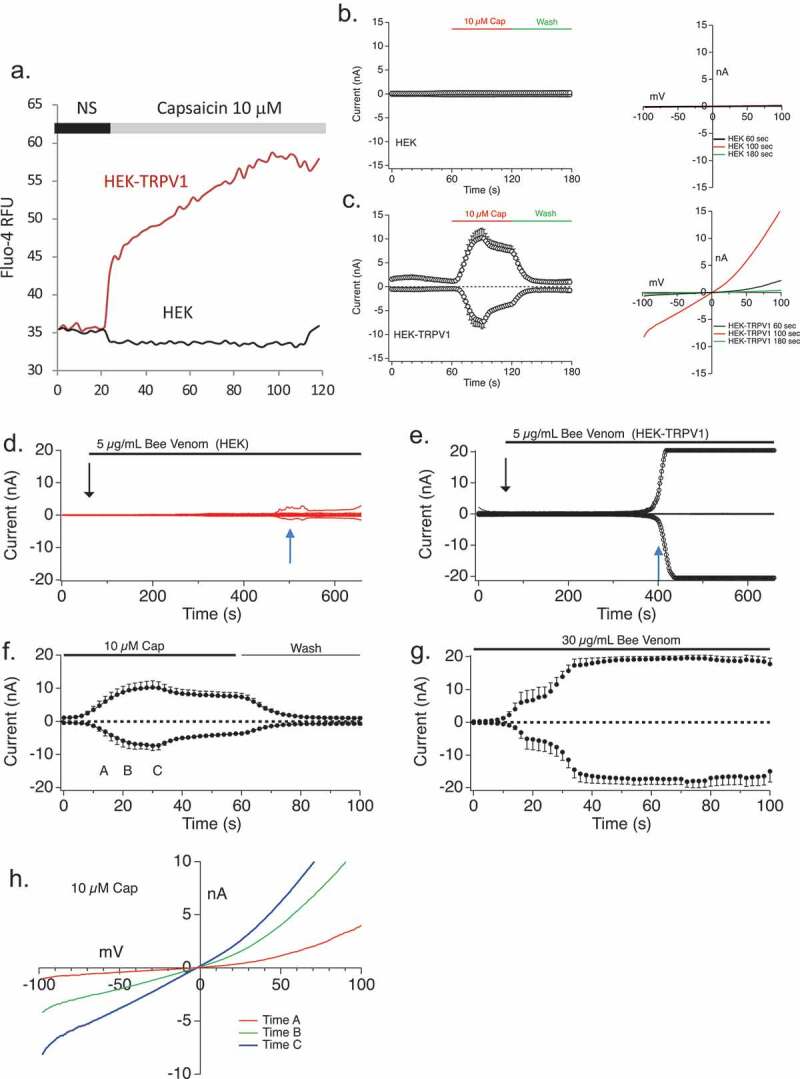
Relationship between TRPV1 and Bee Venom-induced currents. (a) HEK wild-type cells and TRPV1 overexpressing HEK cells were loaded with Fluo-4 and stimulated with capsaicin in the presence of 1mM external CaCl_2_, showing TRPV1-dependent Ca^2+^ responses conferred by expression of the channel. (b,c) Capsaicin-induced conductance measurements in HEK (b) and HEK-TRPV1 (c). *Left panels* show current development over time. *Right panels* show current–voltage relationships. (d) Current development of HEK wild-type cells measured for 650 s with 600 s of 5 µg/ml Bee Venom external application (n = 5). (e) Current development of TRPV1 overexpressing HEK cells measured for 650 s with 600 s of 5 µg/ml bee venom external application (n = 4). Black downward arrows in D, E represent time of addition of stimulus. Blue upward arrows indicate time of extraction of I/V relationship shown in Figure 4(m,n.f). Current development of TRPV1 overexpressing HEK cells measured for 650 s with 600 s of 10µM Capsaicin external application (n = 4). (g) Current development of TRPV1 overexpressing HEK cells measured for 650 s with 600 s of 30 µg/ml bee venom external application (n = 4). The current development graphs were generated by extracting currents at −80 mV and +80 mV. (h) Current-voltage relationship for TRPV1 stimulated by capsaicin. I/V curves were extracted at indicated time points in Figure 4(f). (i,j) Current-voltage relationship for conductance stimulated by Bee Venom. Current development over time is shown in (i), and I/V curves (j) were extracted at indicated time points in Figure 4(f). (k,l) Current development (k) and I/V relationships (l) of Bee Venom-induced conductances in HEK TRPV1 with sodium (Na^+^) or N-methyl D Glucosamine (NMDG) as the primary current carrier in the external solution. The current development graphs were generated by extracting currents at −80 mV and +80 mV. (m,n) Extracted I/V curves from Figure 4 D, E at times indicated by blue upward arrows, showing a lack of CRAC-like rectification and E_rev_ in the small HEK-WT conductance.

**Figure 4.**
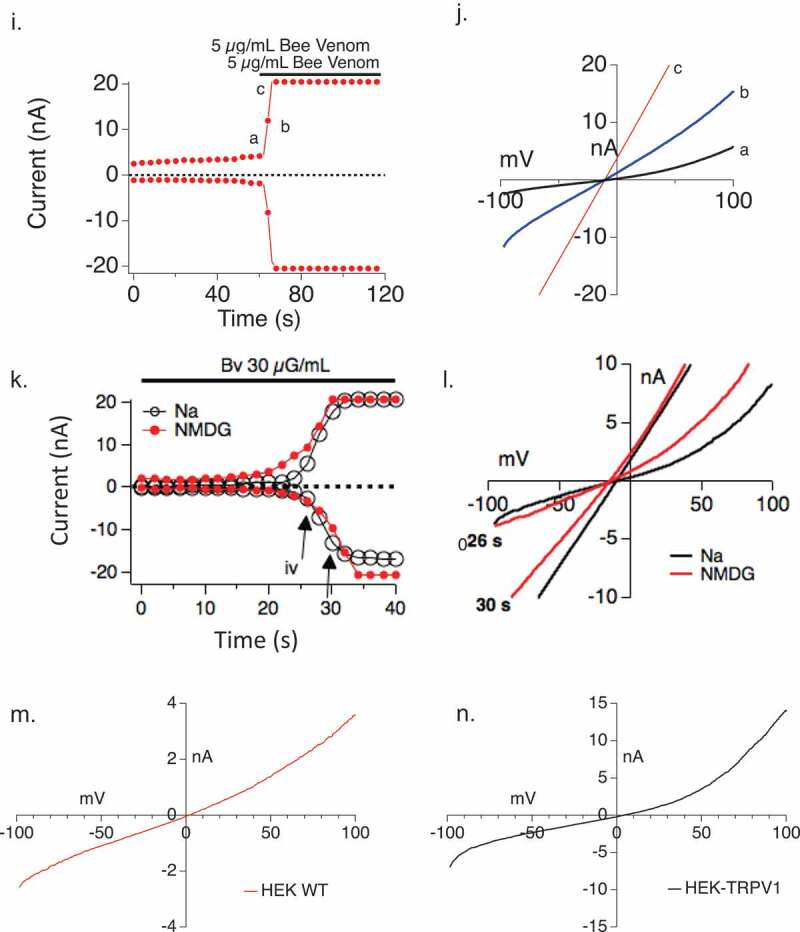
(Continued).

While initial TRPV1 currents are outwardly rectifying, these currents rapidly linearize to a non-rectified conductance and can be inactivated by washout (Figure 4(f,h)). These data are consistent with the established two state nature (normal and pore-dilated) of TRPV1 [33,34]. Figure 4(i–l) examines the dilation state of BV-induced TRPV1. As for Capsaicin, the BV-induced current rapidly linearizes and decreases in rectification. Low doses of BV can distinguish these two states but higher doses cannot. This is further evidenced in Figure 4(k), which shows that the conductance induced by BV is equally permeant to sodium and the large cation NMDG (N-methyl D-Glucamine). Permeation by NMDG is a hallmark of the dilated state of TRPV1 [35,36].

In WT cells (Figure 4(d)) a very small conductance was evoked by bee venom. We initially assumed this would represent the very small CRAC current that is present in HEK, but the extracted I/V curve (Figure 4(m,n)) is inconsistent with CRAC and may hint at a non-TRPV1 TRPV-family member such as TRPV2. We and others have exhaustively demonstrated that there is no endogenous TRPV1 in HEK (measured by response to Capsaicin), and indeed the extracted I/V in the HEK-TRPV1 does show a different rectification profile to that in the WT cells (Figure 4(m,n)).

**Figure 5.**
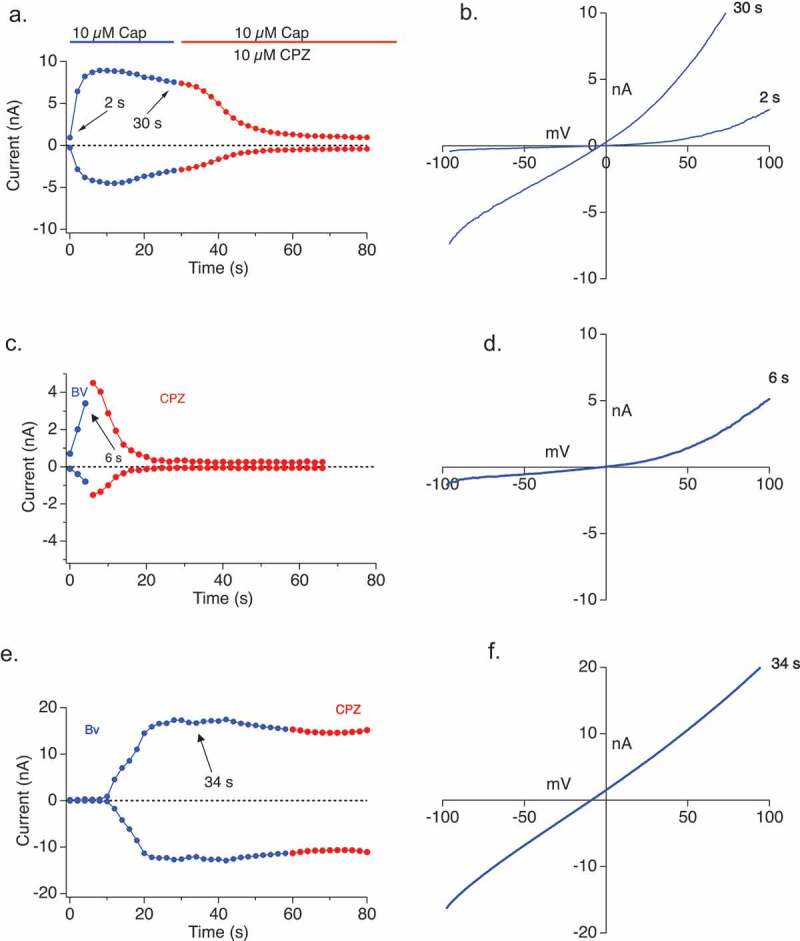
Bee Venom application elicits Capsazepine-sensitive and pore-dilating TRPV1 currents in TRPV1 over-expressing HEK cells. (a,b) Current development (a) and I/V relationship (b) of TRPV1 overexpressing HEK cells with 10 µM Capsaicin application (blue) and followed by 10 µM Capsazepine application (red) (n = 3). (c,d) Current development (c) and I/V relationship (d) of TRPV1 overexpressing HEK cells after 30 µg/ml bee venom application (blue) and 10 µM Capsazepine application (red) with the TRPV1 inhibitor added at 10 sec. (f,f) Current development (e) and I/V relationship (f) of TRPV1 overexpressing HEK cells after 30 µg/ml bee venom application (blue) and 10 µM Capsazepine application (red) with the TRPV1 inhibitor added at 60 sec. The current development graphs were generated by extracting currents at −80 mV and +80 mV.

We noted linearization of the I/V curve in response to bee venom (Figure 4(i,j)), and postulated that might represent either an attainment of the pore-dilated state of TRPV1 or that bee venom is initiating a different type of conductance at later time points due to some hemolytic activity (note though that cell viability remains high throughout the measurement period). We, therefore, applied a TRPV1 inhibitor to differentiate between these possibilities. Capsazepine was applied to Capsaicin-activated current, as a positive control, in the HEK-TRPV1 cells and the current decreased (Figure 5(a,b)). When applied to bee venom-elicited currents prior to any state transition or linearization, capsazepine caused a significant decrease in the current amplitude (Figure 5(c,d)). In contrast, when applied after the state transition or linearization has occurred (Figure 5(e,f)), capsazepine was without effect on the current amplitude. Overall, we observe both state 1 (non-dilated) and state 2 (pore-dilated) TRPV1 currents in response to BV, but also note a third manifestation in some cells where a very late developing large amplitude V1 (or possibly leak, not shown) appears that is also insensitive to the TRPV1 antagonist capsazepine.

### Individual venom component mimetics activate different conductances in model mast cells

**Figure 6.**
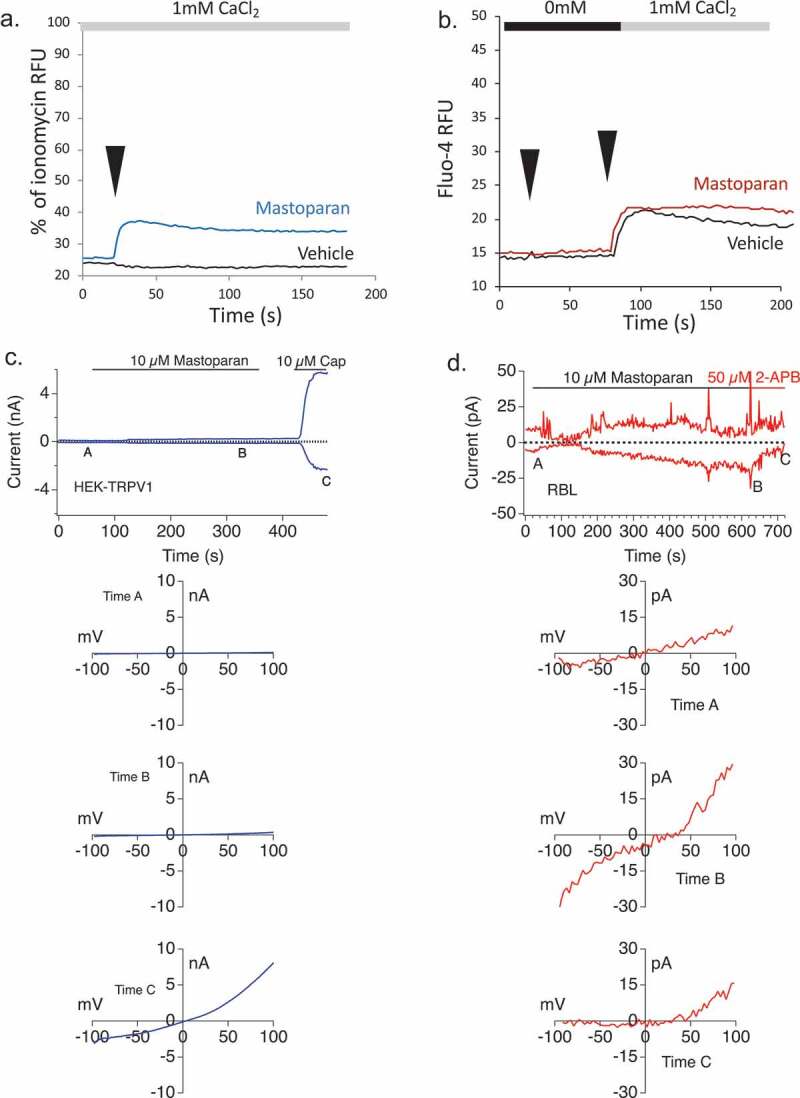
(a) Mastoparan application in RBL-2H3 and TRPV1 overexpression HEK cells (a) Fluo-4-AM was used to load RBL2H3 in a modified Ringer’s solution containing 1 mM external CaCl_2_. Experimental readings were carried out either in 1mM external CaCl_2_ (a) or in 0mM CaCl_2_ with 1mM EGTA followed by readdition of CaCl_2_ to a concentration of 1mM final. (B) Cells were stimulated after a period of establishing baseline (usually 20 s) by addition of vehicle (matched diluent to each stimulus), or 10 μM Mastoparan. Note that “vehicle” effect in B likely represents passive store depletion opening I_CRAC_ channels rather than a Mastoparan effect. (c) *Upper panel*. Current development of a representative TRPV1 overexpressing HEK cell extracted at −80 mV and +80 mV over 480 s of recording after 300 s of 10 µM Mastoparan external application and 50 s of 10 µM Capsaicin application. *Lower panels*. Current-voltage relationships at indicated times in the current development from Figure 6(c). (d) *Upper panel*. Current development of a representative RBL2H3 cell extracted at −80 mV and +80 mV over 700 s of recording after 600 s of 10 µM Mastoparan external application and then application of 2-APB. *Lower panels*. Current-voltage relationships at indicated times in the current development from Figure 6(d). The current development graphs were generated by extracting currents at −80 mV and +80 mV.

The calcium assay data above suggest that several individual components that are found in the venom can cause calcium entry. We, therefore, explored whether the observed conductances in response to the whole venom could reflect conductances induced by individual components. Antigenic cross-linking of FcεRI has been exhaustively shown to activate the CRAC current, which is not explored further here [37–40]. We focused upon Mastoparan, Arachidonic Acid, and Mellitin. In population-based Ca^2+^ assays, Mastoparan elicited calcium influx (Figure 6(a)) but no appreciable store release in the bulk assay (Figure 6(b)), although at the single-cell level, we record a small percentage of cells that do manifest store release in response to Mastoparan (not shown). Mastoparan was tested to see if it elicited a TRPV-like conductance and neither the I/V relationship nor the current development responses in HEK-TRPV1 challenged with Mastoparan support significant activity at TRPV1 (Figure 6(c)). In RBL2H3 cells we found that 10 µM Mastoparan induced a CRAC-like current after leak subtraction that showed inward rectification with a reverse potential around +45 mV (Figure 6(c,d)). The Mastoparan induced current in the RBL2H3 cells was sensitive to 50 µM 2-APB and showed current inhibition similar to CRAC currents (Figure 6(d)). We noted that compound 48/80 (a synthetic secretagogue) also activated a CRAC-like current, which was inhibited by 2-APB (data not shown).

**Figure 7.**
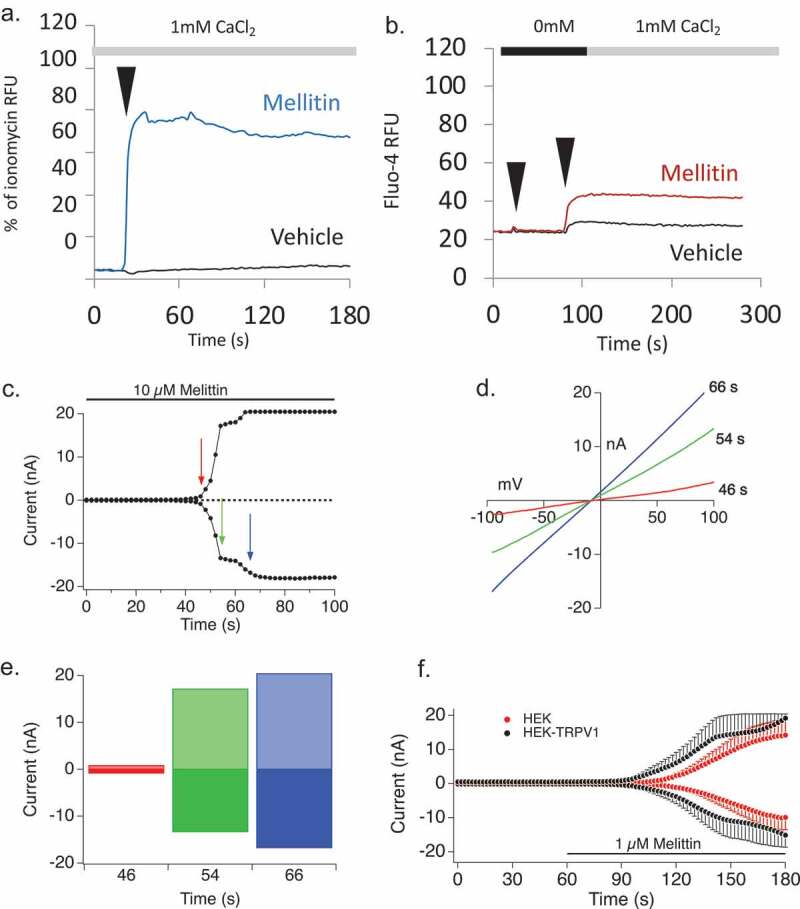
Melittin-induced Ca^2+^ responses and conductances. (a,b). Fluo-4AM was used to load RBL2H3 in a modified Ringer’s solution containing 1 mM external CaCl_2_. Experimental readings were carried out either in 1mM external CaCl_2_ (a) or in 0mM CaCl_2_ with 1mM EGTA followed by re-addition of CaCl_2_ to a concentration of 1mM final (b). Cells were stimulated after a period of establishing baseline (usually 20 s) by addition of vehicle (matched diluent to each stimulus), or 1 μM Mellitin. (c,d) Current development graph, extracted at −80 and +80 mV, (c) and I/V relationships (d) resulting from 10 μM Mellitin stimulation of RBL2H3. (e) Histogram representation of rectification characteristics of the conductances measured at the indicated time points in Figure 7(c,d). (f) Current development graph of HEK WT and HEK-TRPV1 stimulated with Mellitin. (g,h) Sensitivity of Mellitin-induced Ca^2+^ responses to TRPV1 inhibitors in HEK-TRPV1. Fluo-4-AM was used to load RBL2H3 in a modified Ringer’s solution containing 1 mM external CaCl_2_. Experimental readings were carried out in 1mM external CaCl_2._ Capsaicin (1 μM) (g) or Mellitin (10 μM) (h) were applied at 20 sec. After recording for a further 50 s, an inhibitor cocktail of 10 μM each of CPZ and BCTC was added. I, J. Sensitivity of Mellitin-induced Ca^2+^ responses to TRPV1 inhibitors in RBL2H3. Fluo-4-AM was used to load RBL2H3 in a modified Ringer’s solution containing 1 mM external CaCl_2_. Experimental readings were carried out in 1mM external CaCl_2._ Application sequences were Mellitin (20 s), followed by vehicle or an inhibitor cocktail of 10 μM each of CPZ and BCTC (40 s) (i), or vehicle followed by vehicle or the inhibitor cocktail (j). (k) Baseline (10 s timepoint), peak (25 s timepoint) or final attained (180 s timepoint) levels of Ca^2+^ signal were plotted for RBL2H3 treated with Mellitin (at 20 s) then vehicle (at 60 s), blue bars; Mellitin (at 20 s) then CPZ/BCTC (at 60 s), red bars; or vehicle (at 20 s) then CPZ/BCTC (at 60 s), green bars.

Analysis of Mellitin, a bee venom component, revealed a complex picture. Mellitin (1 μM) elicited calcium influx (Figure 7(a)) but no appreciable store release (Figure 7(b)) in RBL2H3. Mellitin-induced currents in RBL2H3 are TRPV-like (Figure 7(c)) rather than CRAC- or ARC-like in their I/V relationship (Figure 7(d)), and there has been a published study in sensory neurons suggesting Mellitin activation of TRPV1 [41]. However, these current rapidly lose their slight rectification (Figure 7(d,e)), and they could represent a pore-forming activity of Mellitin itself which would presumably manifest as a high I_max_, with a highly linear I/V, conductance [42,43]. Figure 7(f) shows that Mellitin is active both in HEK WT and TRPV1-expressing cells, suggesting that much of its activity is not dependent on the presence of TRPV1 protein. However, there is a slight difference between WT and TRPV1-expressing cells, in that current appears earlier in the TRPV1 containing cells and attains a slightly higher I_max_ more rapidly (Figure 7(f)). We asked if inhibition of TRPV1 had any impact on the Ca^2+^ responses initiated by Mellitin. In bulk calcium assays in HEK-TRPV1, we see Mellitin behave as a pore-former (note rapid decline in fluorescence likely implies leaching of Ca^2+^ dye). However, this pore-forming activity is partially sensitive to a cocktail of two different modality TRPV1 inhibitors, Capsazepine (CPZ) and BCTC (N-(4-tert-Butylphenyl)-4-(3-chloropyridin-2-yl)piperazine-1-carboxamide) (Figure 7(h)) which affect Capsaicin-induced responses as would be expected (Figure 7(g)). In mast cells, we see the third scenario. Here, we note that there is less pore-forming activity (evidence of rapid dye-leaching) in response to 10 μM Mellitin (*cf* Figure 7(i,h)) no effect of CPZ/BCTC (Figure 7(i,j,k)). Thus in HEK TRPV1, the effect of Mellitin appears primarily pore-forming with some contribution from a CPZ/BCTC-sensitive TRPV1. In RBL2H3 the cells are more resistant to the pore-forming activity of Mellitin (perhaps an immunocyte adaptation) and CPZ/BCTC are not impacting the Ca^2+^ entry. There are also marked dose-dependent differences since at 1 μM Mellitin neither HEK-TRPV1 or RBL2H3 show the dye-leaching that we think indicates pro-forming activity of Mellitin.

### Spatial analysis of response frequencies in populations of cells exposed to venom or its components

**Figure 8.**
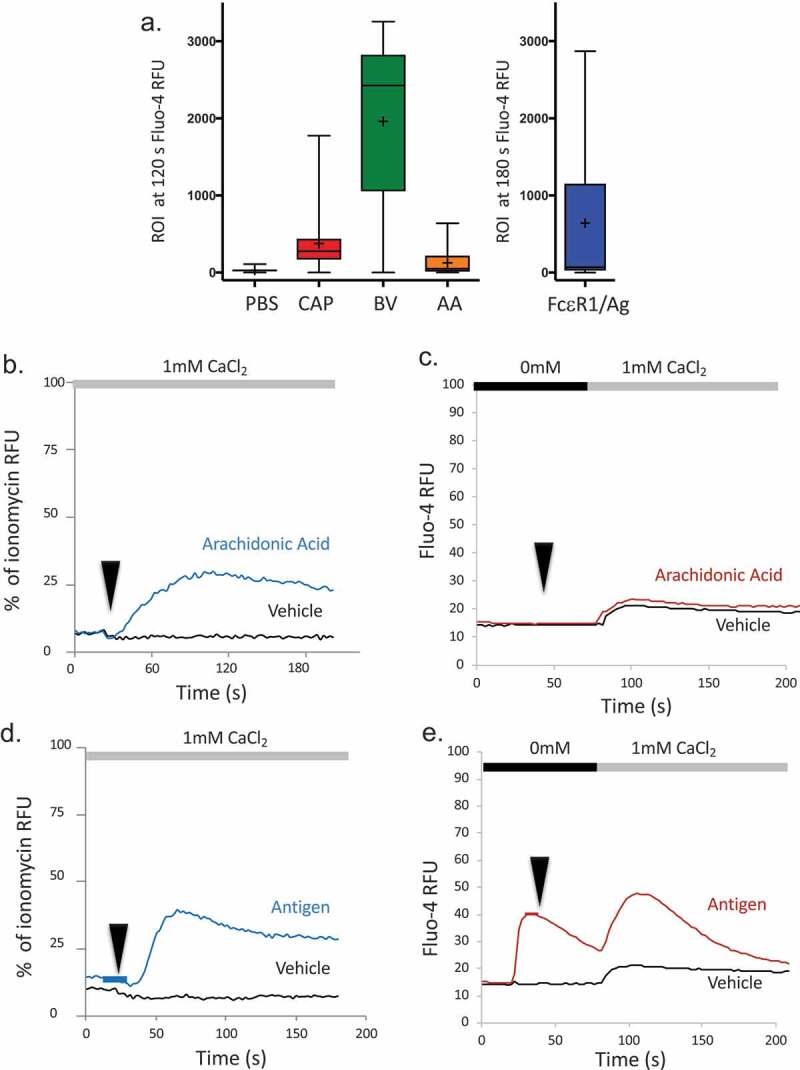
Frequency of RBL2H3 activation by venom and related stimuli. (a) RBL2H3 were loaded with Fluo-4 and stimulated as indicated. Fields of cells were imaged using confocal imaging and regions of interest (ROI) were drawn (Nikon NIS Elements) around the circumference of each cell at a *z* layers positioned at 25%, 50%, and 75% of the measured height of the cell. Changes in Fluo-4 intensity over time were plotted for each ROI, averaged between the *z* discs, and a box plot generated for the minimum, maximum, median and 25th and 75th percentiles of attained fluorescence intensity in that ROI at 120 s (100 s after application of stimulus), or 180 s for FcεRI/antigen (also 100 s after application of stimulus). Numbers of ROI analyzed were PBS (n = 74), Capsaicin (n = 121), Bee venom (n = 160), Arachidonic acid (n = 74), FcεRI/antigen (n = 71). (b–e) Population-based responses to Arachidonic Acid, and FcεRI/antigen in the absence and presence of external Ca^2+^, shown in comparison to Figures 2(a,b); 6(a,b), and 7(a, b).

Population-based bulk calcium responses are recorded for ~300,000 cells per datapoint. These types of data tell us little about the proportion of cells in a population that are responding and driving the bulk calcium signal. The current surveys and subsequent data, on the other hand, suggest that single cells respond to Bee Venom in a differentiated fashion, i.e. one type of conductance predominates. We performed confocal imaging analysis of calcium signals across a population of RBL2H3. We examined fields of cells using confocal imaging of calcium fluxes and produced a frequency analysis of Fluo-4 fluorescence intensity at a 120-s benchmark timepoint, representing the sustained phase of a cell’s calcium signaling response (Figure 8(a)). Regions of interest for each cell (ROI) were drawn at the interface with the coverslip in order to capture the area of the whole cell, and each cell’s time series was averaged from 3 z discs at 25%, 50% and 75% of the cell’s total height. Figure 8(a) summarizes the responses to the various stimuli and compares them to the population-based assays (Figures 8(b–e) and 2(a,b)). While responses to arachidonic acid are rarest, most cells respond to bee venom or secretagogue, and capsaicin and antigen/FcεRI are intermediate stimuli in terms of frequency of cell responses.

**Figure 9.**
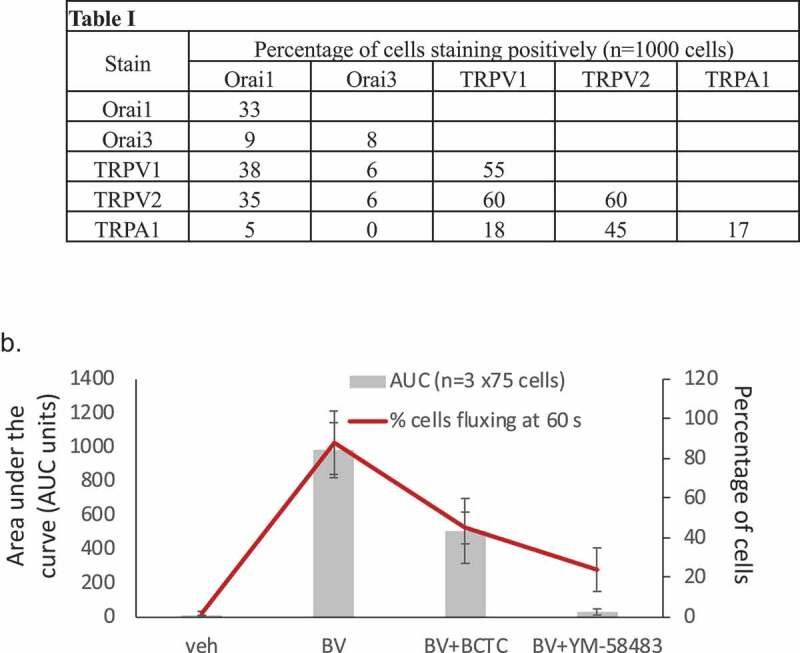
Table 1 Frequency analysis of anti-ion channel immunofluorescence staining in RBL2H3. RBL2H3 were stained with the indicated antibodies alone or pairwise. After mounting, imaging was performed using epifluorescence and 1000 cells were scored for positive or negative membrane-localized staining for the indicated ion channel. Background subtraction was performed using instrument gains set on the basis of the secondary antibody alone control. Positive staining was defined as membrane positivity at 50% or greater of the maximal fluorescence signal for a given cell and color line. (b) Impact of TRPV1 and CRAC inhibitors on population-based calcium responses and frequency of those responses in RBL2H3. Ca^2+^ imaging was conducted as described for Figure 8 in the presence of vehicle, 30 μM Bee Venom or 30 μM Bee Venom with the addition of either 10 μM BCTC or 10 μM of the CRAC inhibitor YM-58,483. Area under the curve (AUC) or response frequency counts were then performed.

Using immunocytochemistry (Figure 9, Table 1), we could not find strong evidence of restricted channel expression (i.e. that individual cells expressed only one type of channel, CRAC, ARC or V1, for example). Certainly, many cells in populations of RBL2H3 co-stained positively with antibodies to two of the channels. These data suggest that the dominance of one conductance in a particular cell evidenced by the current survey in Figure 2 may not be explained simply by restricted expression of one channel type per cell. Figure 9(e) shows that, using confocal imaging, we looked at the impact of BCTC (TRPV1 inhibitor) and the CRAC inhibitor YM-58,483 on the population-based measured flux (area under the curve, AUC, of total cell population) and the number of cells contributing to that flux (% of the cells in the population that respond). There is a disconnect between these two measures, indicating that small numbers of cell can contribute disproportionately to measured population-based fluxes. These data also reinforce that inhibition of any one ion channel that is activated in response to BV may not be sufficient for therapeutic purposes.

#### Summary

Table II in Figure 10 offers a summary of the data presented in this study. Figure 10 summarizes the pathways initiated downstream of venom exposure identified in this study.

**Figure 10.**
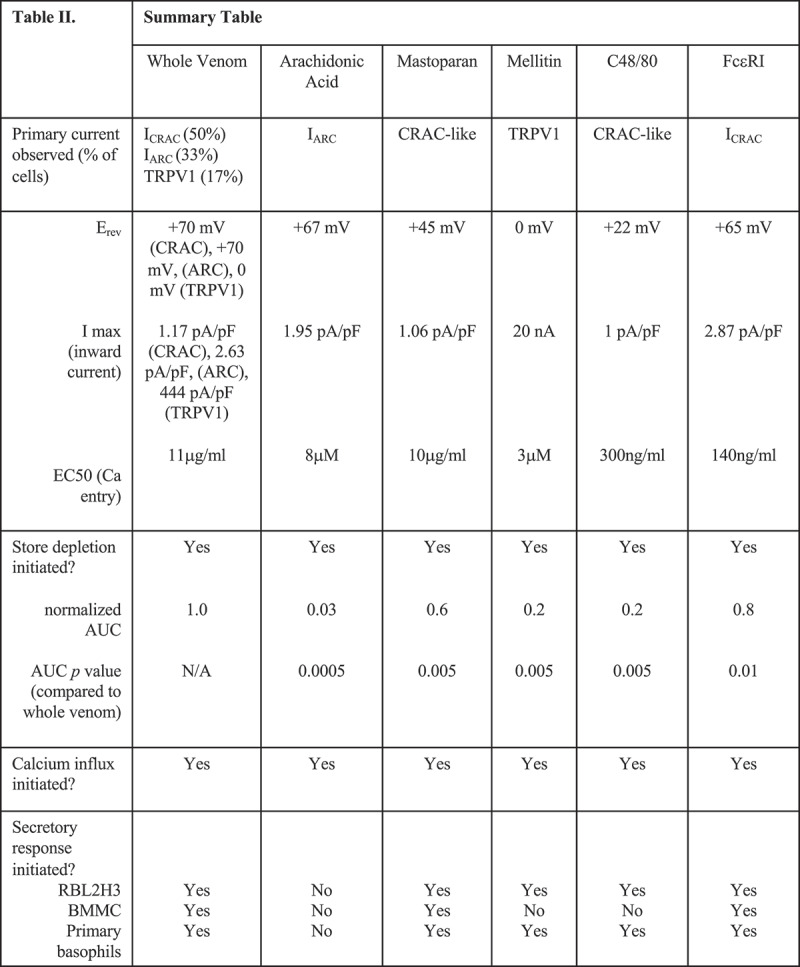
Table 2 Summary of data presented in this study. Tabulation of the primary-observed current, its physiological characteristics and downstream effects on calcium release, Ca^2+^ entry and secretory responses, in response to the whole venom of the indicated components. (a) Schematic summary of putative venom-induced ionotropic pathways in mast cells. Both venom itself (via its intrinsic peptide and small-molecule secretagogue components) and the physicochemical changes in tissue that are initiated by envenomation have the potential to activate TRPV subfamily non-selective cation channels. Venom PLA2 can generate AA, which has the capacity to activate ARC, and there are well-established pathways for antigenic activation of the CRAC pathway. Possible additional linkages are denoted by pink arrows. GPCR, G-protein-coupled receptor; TRP, Transient Receptor Potential; AA, Arachidonic Acid; ARC, Arachidonic acid Activated current; SERCA, Sarcoplasmic-Endoplasmic Reticulum Ca^2+^-ATPase; Thaps, Thapsigargin; InsP3, inositol 1,4,5, trisphosphate; CRAC, Ca^2+^-Release Activated Current; ORAI1, calcium selective ion channel encoded in humans by the ORAI1 gene; FcεRI, high affinity receptor for immunoglobulin E; IgE, immunoglobulin E.

**Figure 10.**
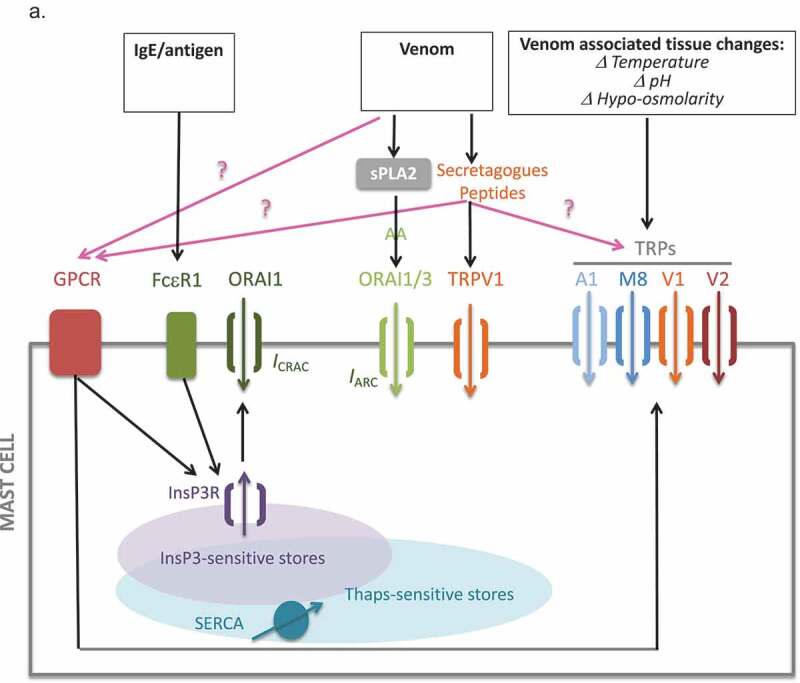
(Continued).

## Discussion

Mast cells respond to a strikingly diverse array of stimuli, ranging from immunological challenges, to environmental and physiological small molecules, to physicochemical and mechanical inputs. These stimuli may be experienced by the cell as single inputs, or in the context of complex mixtures such as venoms which contain multiple potentially activatory factors, or as co-incident inputs resulting from parallel simultaneous exposures. Ion channels, and especially calcium channels, are central examples of proteins that broker cellular responses to each of the stimulus categories above, and mast cells clearly co-express multiple calcium channels. A central question of this paper was to begin decoding this complexity, asking how cells respond to complex stimuli at the single cell and population level, which channels are players in the responses, and what happens functionally and in terms of calcium signaling when co-incident stimuli are received that are capable of activating several signaling pathways in parallel. The key findings (see Table 2) of the current paper are (1) that bee venom contains components that activate multiple Ca^2+^ -permeant ion channels in mast cells including (but not limited to) TRPV, CRAC and ARC conductances, (2) that activation of these conductances can be attributed to specific venom components or their mimetics, and (3) that population-based measurements do not reflect the single-cell heterogeneity of responses, where application of the pluripotent stimuli to cells that co-express multiple ion channel species tends to lead to the measurement of one dominant conductance per cell. The first and second observations have implications for therapeutic targeting of ion channels to suppress unwanted responses to venom and for the informed design of therapeutic *Hymenoptera*-based therapies [44–50] for cancer, pain, and inflammation. The third of these key findings is intriguing and suggests a novel mechanism by which cellular decisions to activate one ion channel rather than another, despite co-expression, for future investigation.

We performed a current survey of a model mast cell line selected for its pleiotropic responsiveness to multiple pro-inflammatory inputs, the RBL2H3. Future studies will need to replicate these experiments in cells such as peripheral blood basophils, since tissue-isolated primary mast cells may offer prohibitive technical challenges. We initially focused on only three calcium entry mechanisms, which significantly underestimates the true complexity of the system, since other TRPV, TRPA, TRPC, TRPM, and other calcium permeant conductances are likely to be present in this system [51–54]. Our data show that *I*_CRAC_, *I*_ARC_ and TRPV conductances are present in model mast cell populations, but at varying frequencies and abundancies, and responding to diverse single stimuli. Heterogeneous stimuli such as *Hymenoptera* venom may activate multiple classes of conductance at the population level but tend to lead to the measurement of only one type of conductance per cell. Having identified interesting findings in this cell line, we will extend our findings to primary bone marrow-derived mast cells and human blood basophils. Preliminary data (not shown) from these studies suggest that the immature bone marrow-derived mast cells are more CRAC-dominant, whereas basophils have wider representations of ARC and TRP responses similar to RBL2H3. Limitations on our ability to isolate tissue-conditioned and resident mast cells make it difficult to assess whether the diversity of expression of conductances is related to maturation or conditioning of distinct mast cell subsets.

The responses to the whole venom suggest that in individual cells are programmed to respond primarily via one conductance pathway, despite the fact that with venom application they are coincidently experiencing stimuli that can target multiple conductances. Venom exposure results in the development of CRAC-like, ARC-like, and TRPV-like currents. Our immunofluorescence data suggest that this is not solely reflecting the differential expression of channel proteins, in that we see many cells that immunodecorated with antibodies against two or more of the channel species studied here. Thus, the mechanism by which one pathway gains dominance in cells that express the machinery for multiple responses is not yet clear but is likely to be complex since other factors such as cell cycle status can impact the representation of channel species in individual cells [55]. It seems likely that in this RBL2H3 system, the population has some pre-existing heterogeneity in either expression of the channels and/or their functionality. If translated to physiological mast cells *in vivo*, this heterogeneity might be reminiscent of the situation in sensory neuron bundles, where individual cells express different combinations of dominant TRPV-family channels to encode differential responses to physicochemical inputs [56,57].

Our study provides some in-depth analysis of responses to venom components or their mimetics. However, while we have studied exemplars of the main *classes* of likely ionotropic stimuli in the complex venom mixture, there may well remain additional stimuli that are coupled to Ca^2+^ entry (e.g. other secretagogue peptides, small molecules such as cyclin nucleotides, other bioactive lipids). We suggest that systematic venom dissection with fractionation coupled to higher throughput electrophysiology would be the next steps in evaluating these possibilities. The secretagogue mastoparan is stimulating broadly CRAC-like currents (highly positive reversal potentials, low amplitude, sensitivity to 2-APB). This is inconsistent with any lytic activity of mastoparan [58], which could result in large linear currents. Rather, in these cells a classic mastoparan mechanism is more likely, direct activation of Gq [59,60] or PLC [61,62] leading to Ins (1,4,5) P3 production and store release followed by CRAC activation [63,64]. In our bulk assays, we do not detect predicted calcium store release in response to mastoparan, possibly due to the sensitivity of the system. The lack of ARC-like currents in response to mastoparan is consistent with the finding of Choi *et al.*, who found that mastoparan does not mobilize AA formation [65].

We also assessed compound 48/80, a mast cell secretagogue often studied in parallel to mastoparan. The currents that respond to compound 48/80 have a more TRP-like I/V curve, [66] and a lack of sensitivity to 2-APB (data not shown). We have not definitively identified them, and our practice of using close to physiological amounts of Mg^2+^ (1 mM compared to physiological levels of ~0.5 mM) in our recording solutions, rather than the high levels of Mg^2+^ needed to block TRPM7, may be leading to TRPM7 presence in these recordings and larger than actual outward currents as a result, so we did not include them in this report. Further work on both Mastoparan and other secretagogues may be of interest: A recent paper suggests that ORAI2 (a CRAC component) augments antigen- and secretagogue-induced calcium responses [37]. Older studies suggest that secretagogues can work via PLD [67], and the PLD product phosphatidic acid (PA) has been linked to potassium channels and TRPV1 (as lyso-PA) [68,69]. More complexity is suggested by a study showing that secretagogue-induced release and influx responses can be differentially reliant on AA signaling [70], and by possible G-protein involvement [71].

Mellitin is a venom component with interesting properties. Mellitin clearly activates TRPV1-dependent responses and has previously been shown to activate V1 in sensory neurons [23]. Mellitin poses several open questions. We have not yet discerned the difference between “pore-forming” activity and the ability to induce the dilated, highly permeant, state of TRPV1 or possibly the activation of TRPV2 or another TRP (given the lack of sensitivity to CPZ/BCTC). At low concentrations, Mellitin can also clearly induce some conductance in the WT HEK cells that do not appear to represent a lytic current, possibly this is TRPV2 or a TRPC conductance [72]. RBL2H3 show a linear TRPV-like current in response to mellitin, which compares closely to either pore-dilated V1 currents induced by mellitin in a TRPV1 over-expression system or to the type of conductance related to the putative pore-forming role of some venom peptide components. Such pore-forming peptides can be lytic or non-lytic, and the apparent health of the cells (no obvious morphological changes, not trypan blue positive) under conditions of venom or mellitin application suggest that direct hemolysis is not occurring during our experimental time period.

In summary, heterogeneous stimuli such as *Hymenoptera* venom may activate multiple classes of conductance at the population level but at the single-cell level initiate single conductance responses that are heterogeneous across the cell lines used here, and appear to be similarly heterogeneous in single-cell calcium assays of primary basophils and mast cells (not shown). The mechanism by which the multi-component venom, at the single-cell level, is somehow “less than the sum of its parts” and selectively activates one conductance type per cell, is of interest to explore in future studies. The relationship between our data and prior studies showing “all or none” single cells responses but graded population responses via CRAC merit further study [40]. Single-cell secretion assays are also required to assess which venom-activated pathways are resulting in functional responses downstream of calcium entry, as not all calcium entry profiles seen here may be sufficient to initiate histamine secretion, for example. Identification of the molecular targets of Bee Venom within the diversity of Ca^2+^ channels, and the functional impact of the resulting Ca^2+^ entry, may inform decisions about which channels should be targeted to mitigate pathological effects of envenomation. Finally, in addition to the presumed mast cell activatory, pro-inflammatory, responses studied here, the implications of bee venom therapeutically as a desensitizing agent for a nociceptive TRP such as TRPV1 should be considered in the documented anti-inflammatory and anti-nociceptive effects of venom [47,73].
